# Response to: Comment on “circSMARCA5 Functions as a Diagnostic and Prognostic Biomarker for Gastric Cancer”

**DOI:** 10.1155/2019/9356804

**Published:** 2019-09-23

**Authors:** Juan Cai, Zhiqiang Chen, Xueliang Zuo

**Affiliations:** ^1^Department of Oncology, The First Affiliated Hospital, Yijishan Hospital of Wannan Medical College, Wuhu, Anhui, China; ^2^Key Laboratory of Non-coding RNA Transformation Research of Anhui Higher Education Institution (Wannan Medical College), Wuhu, Anhui, China; ^3^Hepatobiliary Center, The First Affiliated Hospital of Nanjing Medical University, Key Laboratory of Liver Transplantation, Chinese Academy of Medical Sciences, NHC Key Laboratory of Living Donor Liver Transplantation, Nanjing, Jiangsu, China; ^4^Department of Gastrointestinal Surgery, The First Affiliated Hospital, Yijishan Hospital of Wannan Medical College, Wuhu, Anhui, China

We are very grateful to Dr. Farid Rahimi and Dr. Amin Talebi Bezmin Abadi for their valuable comments and suggestions [1] on our recent article entitled “circSMARCA5 Functions as a Diagnostic and Prognostic Biomarker for Gastric Cancer” [2]. We have perused the comments and hope the provided responses will address the concerns.

As previously reported, the functions of circSMARCA5 in different cancers are discrepant. The expression level of circSMARCA5 is downregulated in most types of cancers, including hepatocellular carcinoma [3, 4], glioblastoma [5, 6], and cervical cancer [7]. In prostate cancer, however, upregulated circSMARCA5 expression level is observed [8]. In our study, we confirmed the downregulation of circSMARCA5 using both gastric cancer (GC) tissue and blood samples. Consistently, all the published studies [3–8] used tissue samples to investigate the dysregulation of circSMARCA5 in cancers. Notably, the increased expression level of circSMARCA5 was also determined using prostate cancer samples and the matched normal prostate tissues [8]. circSMARCA5 is an androgen-induced circRNA in prostate cancer [8], and the activation of androgen receptor pathway is a common driver of prostate cancer progression [9, 10]. Therefore, the discrepancies in circSMARCA5 expression patterns might be attributed to the complexity of molecular events that occurred during the onsets of different types of cancers. GC is an aggressive illness with a dismal survival outcome; thus, exploring potential diagnostic and prognostic biomarkers is of great importance. For the diagnostic purpose, noninvasive approaches such as liquid biopsies could be developed utilizing plasma circSMARCA5. Tissue circSMARCA5 level might be more useful for patients undergoing radical surgery to predict the clinical prognosis.

With the advances in high-throughput omics technologies, the identification of promising biomarkers has substantially accelerated during the last decades. Biomarker development entails multiple phases, from the discovery of candidate molecules, to validation with training and verification cohorts, followed with multicenter independent cohorts, to a biomarker-based randomized clinical trial, and finally to general clinical application [11]. Noncoding RNAs have become a novel field of biomarker development in GC [12–14]. As discussed in the limitations in our study [2], we proposed the potential clinical value of circSMARCA5 for GC based on data from a single medical center with a relatively small sample size. There is still a long way to go in the pursuit of the application of circSMARCA5 in clinical care of GC patients. Future prospective studies shall be performed in more centers and other populations to further evaluate the clinical significance of circSMARCA5 for GC.

As mentioned, it may be better to employ the *Δ*CT method underlying qRT-PCR to control for differences in expression of circRNAs. However, almost all previous reports have used linear RNA, among which GAPDH has been employed most frequently, for circSMARCA5 normalization [3, 5–8]. To avoid discrepancies arising from methodological approaches, we adopted GAPDH to control for differences in circSMARCA5 expression. Although GAPDH can be deregulated in tumor cells, there is still no perfect internal reference that remains totally unchanged in cancer cells. Normalizing circRNA expression to the geometric mean of several housekeeper genes including GAPDH for qRT-PCR may be one potential solution [15]. Nonetheless, numerous studies still use GAPDH as a quantifying guide and control gene for circRNA in cancer [16–20].

circRNA has become a rising star in the field of cancer biology, and increasing studies are aimed at elucidating the molecular mechanisms and clinical values of circRNAs in cancer [21, 22]. It would be promising yet challenging to further investigate the important roles of circRNAs in GC.
